# Individual- and neighborhood-level education influences the effect of obesity on prostate cancer treatment failure after prostatectomy

**DOI:** 10.1007/s10552-015-0628-y

**Published:** 2015-07-14

**Authors:** Charnita Zeigler-Johnson, Knashawn H. Morales, Karen Glanz, Elaine Spangler, Jonathan Mitchell, Timothy R. Rebbeck

**Affiliations:** Division of Population Science, Department of Medical Oncology, Thomas Jefferson University, Philadelphia, PA USA; Center for Clinical Epidemiology and Biostatistics, University of Pennsylvania, Philadelphia, PA USA

**Keywords:** Prostate cancer, Obesity, Education, Cross-level interaction, Neighborhood SES

## Abstract

**Purpose:**

The relationship between obesity and prostate cancer (CaP) treatment failure is complex and may vary by patient- and neighborhood-level educational attainment. We evaluated whether patient- and neighborhood-level education is associated with the effect of obesity on biochemical recurrence.

**Methods:**

Seven hundred and forty-six CaP cases were classified into four groups: Concordant Low–Low: less educated cases (<4 years college) living in a less educated neighborhood (below-median proportion of college-educated residents; *n* = 164); Concordant High–High: highly educated cases (≥4 years college) living in a highly educated neighborhood (above-median proportion of college-educated residents; *n* = 326); Discordant Low–High: less educated cases living in a highly educated neighborhood (*n* = 69); and Discordant High–Low: highly educated cases living in a less educated neighborhood (*n* = 187). Cox regression models were used to examine associations between obesity and biochemical (PSA) failure after prostatectomy stratified by the concordant/discordant groups.

**Results:**

The association of obesity with biochemical failure varied significantly by educational concordance/discordance (*p* = 0.007). Obesity was associated with risk of biochemical failure for less educated cases residing in less educated neighborhoods (HR 3.72, 95 % CI 1.30–10.65). The relationship was not significant for other concordant/discordant groups.

**Conclusions:**

Obesity effects on CaP outcomes vary by multilevel educational discordance/concordance. Strategies to decrease prostate cancer risk of progression may focus on reduction in obesity, particularly for less educated cases residing in less educated neighborhoods.

## Introduction

Prostate cancer is a major public health burden with few confirmed modifiable risk factors. Obesity, a potentially modifiable risk factor, increases the risk of advanced disease at diagnosis and treatment failure [1–5]. Obesity also varies by socioeconomic status (SES). Poorer neighborhoods are more likely to have higher levels of obesity [6, 7]. A relationship between obesity and poor prostate cancer outcomes and recurrence of prostate cancer has been supported by several reports [8–10]. However, the association between obesity and prostate cancer outcomes has been inconsistent [3, 9, 11–13]. Similarly, low neighborhood SES has been correlated with increasing disease rates and poorer health outcomes, including mortality [14–18].

It has been hypothesized that adverse health effects are related to living in a neighborhood with an SES that is discordant with one’s own SES [19]. This phenomenon of SES discordance is also referred to as cross-level interaction [20]. Associations of SES discordance and health outcomes such as mortality, hospitalizations, and alcohol consumption have been reported [20–23]. Reasons for these effects might be that low-SES individuals in high-SES neighborhoods have limited access to resources or less opportunity to maintain healthy lifestyles [20]. Individuals living in SES-discordant situations may experience differences in cancer education, access to care, and feelings of relative deprivation and stress compared with those living in SES-concordant situations [20, 24].

The goal of this study was to describe educational discordance/concordance in a population of CaP cases and evaluate whether associations between obesity and CaP severity are influenced by educational discordance/concordance.

## Methods

### Study Sample

A prospective study design was used to examine the relationship between discordance in educational attainment at CaP diagnosis and biochemical failure after radical prostatectomy. European American (EA) and African-American (AA) CaP cases were recruited at the University of Pennsylvania Health System (UPHS, Philadelphia, PA) via the Study for Clinical Outcomes Risk and Ethnicity (SCORE). All cases seen in these clinics that were newly diagnosed within the previous 12 months with a histologically confirmed primary CaP at any stage and underwent prostatectomy for treatment of their cancer were eligible for participation in this study.

Informed consent was obtained from all individual participants included in the study under a protocol approved by the Institutional Review Board at the University of Pennsylvania. Case status was confirmed by medical records review using a standardized abstraction form. Men were excluded from this study if they reported having exposure to finasteride or dutasteride at any time prior to their CaP diagnosis, were diagnosed more than 12 months prior to the date of study ascertainment, or had ever been diagnosed with cancer at any site (except non-CaP skin cancer) other than their recently diagnosed CaP.

We used patient-level education obtained from questionnaire self-report. Patient-level education was defined as having attended <4 years or ≥4 years of college. Residential addresses of cases were geo-coded to the census tract level with Geographic Information Systems (ArcGIS) technology [25]. We used census tract college education variables from the 2000 US Census to measure neighborhood educational attainment. The median cut-point (37 %) for “percent of census tract residents with college education” was determined for all cases combined. Because 68 % of the SCORE sample was college-educated, we used college as the cut-point for defining educational discordance. Therefore, we evaluated the cross-level effects of having a college education and residing in a higher or lower than average college-educated community. Educational concordance/discordance was defined as (1) less than 4 years of patient college education and residence in a neighborhood with below-median neighborhood college education attainment; (2) less than 4 years of patient college education and above-median neighborhood college education attainment; (3) four or more years of patient college education and below-median neighborhood college educational attainment; and (4) four or more years of patient college education and above-median neighborhood college education attainment. Groups 1 and 4 represent educationally “concordant groups,” while groups 2 and 3 represent educationally “discordant” groups.

### Statistical analysis

Univariate analyses of patient characteristics were evaluated after stratifying on concordance/discordance groups and obesity status. Non-obese was defined as body mass index (BMI) <30 kg/m^2^, and obese was defined as BMI ≥ 30 kg/m^2^. Chi-square tests and Fisher’s exact tests were used to compare discrete variables across obesity groups. Kruskal–Wallis tests were used to compare differences in medians of continuous variables across obesity groups. Patient characteristics measured at diagnosis included tumor grade, with low grade defined as tumor Gleason score of six or below and high grade defined as a tumor score of seven or greater; prostate-specific antigen (PSA), with low-PSA group defined as <10 ng/ml and high-PSA group defined as PSA ≥ 10 ng/ml; and age group < and ≥60 years.

The primary study outcome was biochemical failure after prostatectomy, defined as a PSA greater than or equal to 0.2 ng/dl after primary treatment. Cases were followed for a median 28 months (range = 2–168 months). Cox regression models were adjusted for census tract, tumor grade, PSA at diagnosis, patient race, age, and the obesity–educational concordance/discordance interaction term. Modification of the association between obesity and biochemical failure by education was assessed by an interaction term in the Cox regression model.

## Results

We studied 227 obese and 519 non-obese incident CaP cases recruited into the SCORE study from 1995 to 2011 and followed for biochemical failure after treatment (radical prostatectomy and radiation) for CaP in the UPHS. Cases ranged in age from 39 to 79 years (SD = 6.54). Thirty percent of SCORE cases were obese (Table 1). Sixty-nine percent of SCORE cases were college-educated (71 % non-obese and 63 % obese, *p* = 0.024). As shown in Table 1, the distribution of obesity was significantly different across the concordant/discordant groups ($\chi_3^2 = 15.314$, *p* = 0.002).

**Table 1:** Education discordance groups (patient education–neighborhood education): demographics (*n* = 746)

Group	Patient characteristics	Total sample *n* = 746	Non-obese *n* = 519 (69.6 %)	Obese *n* = 227 (30.4 %)	*p* value for non-obese–obese comparisons
All SCORE prostate cancer cases	Median age (year)	59	59	58	0.268
	% Married	645 (86.7)	454 (87.8)	191 (84.1)	0.174
	% African-American	104 (13.9)	59 (11.4)	45 (19.8)	**0.002**
	% College Education	513 (68.8)	370 (71.3)	143 (63.0)	**0.024**
	% Ever smokers	375 (50.4)	260 (50.2)	115 (50.9)	0.862
	Median BMI (kg/m2)	27.9	26.5	32.5	**<0.001**
	Median PSA ng/ml	5.2	5.3	4.9	**0.006**
	% PSA > 10 ng/ml	83 (11.3)	64 (12.5)	19 (8.4)	0.109
	% High stage (T3, T4)	202 (27.2)	124 (24.0)	78 (34.5)	**0.003**
	% High grade (7+)	348 (46.7)	224 (43.2)	124 (54.6)	**0.004**

		Total *n* = 164	Non-obese *n* = 101 (61.6 %)	Obese *n* = 63 (38.4 %)	*p* value for non-obese–obese comparisons
Concordant Low–Low	Median age (year)	58	58	59	0.626
	% Married	137 (83.5)	84 (83.2)	53 (84.1)	0.174
	% African-American	44 (26.8)	26 (25.7)	18 (28.6)	0.691
	% Ever smokers	100 (61.0)	58 (57.4)	42 (66.7)	0.238
	Median BMI (kg/m2)	28.6	26.5	32.1	**<0.001**
	Median PSA	5.1	5.4	4.8	0.168
	% PSA > 10 ng/ml at diagnosis	19 (11.7)	14 (14.1)	5 (7.9)	0.231
	% High stage (T3, T4)	49 (29.9)	25 (24.8)	24 (38.1)	0.069
	% High grade (7+)	82 (50.0)	51 (50.5)	31 (49.2)	0.872

		Total *n* = 326	Non-obese *n* = 250 (76.7 %)	Obese *n* = 76 (23.3 %)	*p* value for non-obese–obese comparisons
Concordant High–High	Median age (year)	60	60	59	0.199
	% Married	285 (87.4)	223 (89.2)	62 (81.6)	0.079
	% African-American	22 (6.8)	13 (5.2)	9 (11.8)	**0.043**
	% Ever smokers	147 (45.2)	118 (47.4)	29 (38.2)	0.157
	Median BMI (kg/m2)	27.7	26.5	32.7	**<0.001**
	Median PSA ng/ml	5.3	5.4	5.0	**0.044**
	% PSA > 10 ng/ml	39 (12.1)	33 (13.4)	6 (8.0)	0.213
	% High stage (T3, T4)	84 (25.9)	59 (23.7)	25 (33.3)	0.095
	% High grade (7+)	153 (46.9)	112 (44.8)	41 (54.0)	0.162

		Total *n* = 69	Non-obese *n* = 48 (69.6 %)	Obese *n* = 21 (30.4 %)	*p* value for non-obese–obese comparisons
Discordant Low–High (patient level <neighborhood)	Median age (year)	58	58.5	57	0.734
	% Married	63 (94.0)	42 (91.3)	21 (100.0)	0.301*
	% African-American	7 (10.1)	6 (12.5)	1 (4.8)	0.427
	% Ever smokers	46 (66.7)	30 (62.5)	16 (76.2)	0.267
	Median BMI (kg/m2)	27.3	26.4	33.1	**<0.001**
	Median PSA ng/ml	5.1	5.1	5.6	0.809
	% PSA > 10 ng/ml	9 (11.8)	4 (8.3)	4 (20.0)	0.221*
	% High stage (T3, T4)	19 (27.5)	13 (27.1)	6 (28.6)	0.899
	% High grade (7+)	33 (47.8)	21 (43.8)	12 (57.1)	0.305

		Total *n* = 187	Non-obese *n* = 120 (64.2 %)	Obese *n* = 67 (35.8 %)	*p* value for non-obese–obese comparisons
Discordant High–Low (patient level >neighborhood)	Median age (year)	**58**	58	58	0.825
	% Married	160 (85.6)	105 (87.5)	55 (82.1)	0.313
	% African-American	31 (16.6)	14 (11.7)	17 (25.4)	**0.016**
	% Ever smokers	82 (44.1)	54 (45.0)	28 (42.2)	0.735
	Median BMI (kg/m2)	28.3	26.8	32.4	**<0.001**
	Median PSA ng/ml	5.0	5.1	4.9	0.196
	% PSA > 10 ng/ml	17 (9.2)	13 (11.0)	4 (6.0)	0.300
	% High stage (T3, T4)	50 (26.9)	27 (22.7)	23 (34.3)	0.086
	% High grade (7+)	80 (42.8)	40 (33.3)	40 (59.7)	**<0.001**

* *p* value based on Fisher’s exact test when cells had sample size ≤5

Bold values indicate *p* < 0.05

Among all cases, obese men were more likely than non-obese men to be AA (20 vs. 11 %, *p* = 0.002), less likely to have a college education (63 vs. 71 %, *p* = 0.024), presented with higher BMI (26.5 vs. 32.5 kg/m^2^, *p* < 0.001) and lower PSA (4.9 vs. 5.3 ng/ml), and more likely to have higher tumor stage (35 vs. 24 %, *p* = 0.003) and grade (55 vs. 45 %, *p* = 0.004) at CaP diagnosis (Table 1).

Similarly, in stratified analyses, obese cases in the concordant high patient, high neighborhood education group were more likely to be AA (*p* = 0.043) and have lower PSA levels (*p* = 0.044). Obese cases in the discordant high patient, low neighborhood education group also were more likely to be African-American (*p* = 0.016) and have a high tumor grade (*p* < 0.001). BMI was consistently different by obesity status across each of the education concordance/discordance groups (*p* < 0.001).

Obese and non-obese cases in the concordant low patient, low neighborhood education group and the discordant low patient, high neighborhood education group did not differ in other demographic and clinical characteristics.

**Fig. 1:**
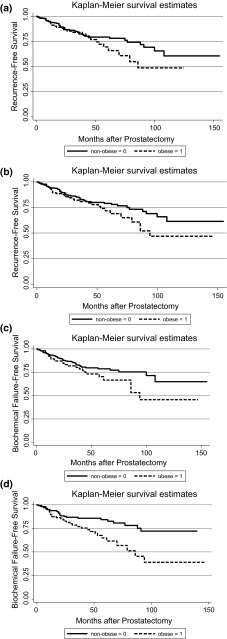
**a** Time to biochemical failure (Recurrence) by obesity: lower patient education, lower neighborhood education. **b** Time to biochemical failure (recurrence) by obesity: lower patient education, higher neighborhood education. **c** Time to biochemical failure (recurrence) by obesity: higher patient education, lower neighborhood education. **d** Time to biochemical failure (recurrence) by obesity: higher patient education, higher neighborhood education

Overall, 78 (10.5 %) patients experienced biochemical failure during the study. Univariately, there were no differences in overall failure rates by obesity status (*p* = 0.068) or concordance/discordance groups (*p* = 0.827). However, obese cases in each concordant/discordant education group experienced a quicker time to biochemical failure (CaP recurrence) than non-obese cases (Fig. 1a–d). In multivariable models, the association of obesity with biochemical failure varied significantly by educational concordance/discordance (*p* = 0.007). Obesity was significantly associated with risk of biochemical failure in the concordant low patient, low neighborhood education group (HR 3.72, 95 % CI 1.30–10.65, Table 2). The HR was similar for the discordant low patient, high neighborhood education group, but the effect was not significant (HR 3.98, 95 % CI 0.60–26.54).

**Table 2:** SCORE models: relationship between obesity and prostate cancer outcomes by college concordance/discordance (patient college–neighborhood college)

Concordance/discordance group	HR for the effect of obesity on biochemical failure (95 % CI)
Concordant—Low–Low	**3**.**72** (**1**.**30**–**10**.**65**)
Discordant–patient level < neighborhood	3.98 (0.60–26.54)
Discordant–patient leve l> neighborhood	1.06 (0.43–2.62)
Concordant–High–High	0.52 (0.17–1.59)

Cox models adjusted for census tract, tumor grade, PSA at diagnosis, patient race, age, and obesity–college concordance/discordance interaction

Bold values indicate *p* < 0.05

## Discussion

Our results suggest that cases of lower education living in concordant lower education neighborhoods are at the highest risk of biochemical failure if they are obese. Elevated BMI previously has been associated with increased risk of biochemical failure [1, 9, 10, 26]. While the literature on SES discordance suggests that low-SES individuals living in high-SES neighborhoods may be at greatest risk of poor health outcomes or mortality, our results of a hospital-based CaP sample show that only obese cases living in a concordant low patient–low neighborhood education context are at particular risk of biochemical failure.

We hypothesize that obese low-education individuals living in low-education neighborhoods may experience “double jeopardy” because they are predisposed to multiple disadvantages at both the individual and neighborhood levels [22]. For CaP, living in education discordant or concordant high education contexts may neutralize risk of biochemical failure in obese cases. Similarly, either higher patient or neighborhood education may buffer risks that may impact poor CaP outcomes.

Several studies have examined SES cross-level effects on various health outcomes. Most have suggested that SES discordance resulted in worse health outcomes. Winkleby et al. [20] reported relationships between SES discordance and overall mortality. Death rates among low-SES men and women were highest in high-SES neighborhoods, lower in moderate-SES neighborhoods, and lowest in low-SES neighborhoods. Taylor et al. [21] extended this work to find similar associations for SES discordance. Higher hospitalization rates were observed among low-SES cases living in high-SES neighborhoods. Mulia et al. [22] reported relationships between SES discordance and high alcohol consumption, also among low-SES individuals residing in high-SES neighborhoods. Similar to our findings with biochemical failure, in a study of breast cancer cases, women with concordant low patient education and low neighborhood SES were determined to have worse all-cause survival than concordant high-SES women [23]. However, the risk was also higher among discordant SES groups (high education and low neighborhood SES) if the cases were AA or Asian American. Our study is the first to examine cross-level effects of education and obesity on CaP outcomes. While other studies defined patient-level SES as education with or without income and defined neighborhood SES as a composite variable to examine cross-level effects, we focused on education discordance because of limited income data at the patient level and the fact that education may be the most valid SES factor for this patient population.

We also examined the separate interactions of patient- and neighborhood-level education with obesity on biochemical failure. Although an increased risk for obese patients with low patient-level education was observed in those separate analyses, our study adds to the CaP literature the finding that the context in which high-risk patients live also matters. We found that only obese patients with low patient-level education living in low-education neighborhoods were at significantly increased risk of biochemical failure.

Obesity is a potentially modifiable risk factor for disease progression and poor outcomes for numerous diseases including prostate cancer. Obesity is believed to increase the risk of advanced tumor stage and grade at diagnosis, younger age at diagnosis, and biochemical failure (disease recurrence) after treatment [1–4]. However, the relationship between obesity and prostate cancer is complex. A recent large cohort study demonstrated that obesity was associated with a decreased risk of low-grade CaP but an increased risk of high-grade CaP [5]. While some previous studies did not support a relationship of obesity with CaP [3, 9, 11–13] or associations with some outcomes and not others [12], inconsistent findings may have been caused by differences in the composition of study populations, including the prevalence of obesity, ethnic distribution, nationality of the population, PSA screening recommendations in international studies, and diagnostic obstacles associated with obesity [3, 12, 27–31].

Both treatment effects and biological effects have been proposed as explanations for the effect of obesity on CaP outcomes [5, 32–35]. After prostatectomy, overweight and obese cases have significantly longer hospital stays compared with normal weight cases. Estimated blood loss during the procedure is also greater in obese and overweight cases [26]. However, potency and continence rates after treatment are similar among weight groups, so technically inferior operations do not account fully for differences in treatment failure [26]. In addition to treatment effects, numerous biological pathways have been associated with dysregulation among obese individuals, including aberrant hormone production, hormone metabolism, and alterations in insulin, insulin-like growth factor 1 (IGF-1), and leptin are well established [36–38]. Obesity also appears to promote hyperandrogenicity and presents a chronic inflammatory environment that sets the stage for cancer progression and poor prognosis, although underlying mechanisms within the tumor are poorly understood [38–42].

We focused on education as a surrogate for SES in this study, but we realize that other neighborhood and patient characteristics may contribute to the associations that we observed. Education and income (and combined metrics including both) are commonly used in the USA as measures of patient- and neighborhood-level SES [20, 22, 23]. Education is readily available in research databases and has been used in other studies of cancer outcomes [23, 43]. Education is also important because it may correlate with knowledge, literacy, sense of empowerment, and skill sets that may be needed to navigate health care, decision making, and coping with disease. Although patient education is not an optimal proxy for individual SES (i.e., the same educational attainment does not result in the same societal advantage for all people or cultures), it is perhaps the best indicator to use in a population of aging men [43]. Many older men live on fixed incomes that are not reflective of their SES or accumulated wealth. Also, poor health may decrease income, but will not alter educational attainment [44]. Unlike income and occupation, educational attainment is often fixed early in adulthood and is less likely than income to be affected by factors such as illness, change in job, or retirement [22]. Education is also predictive of having a more favorable occupation, income, or neighborhood.

Disease risks at the individual and neighborhood level often are impacted by education. According to SEER data, higher educational attainment has been associated with greater risk of prostate and breast cancers alike. Compared to college-educated men, men with less than a college education were 0.79 as likely to be diagnosed with prostate cancer [45]. Prostate screening (and therefore CaP incidence) is more common in men with higher education, white-collar jobs, access to good health care, urban residences, and higher household income [46]. Neighborhood-level education also predicts metabolic syndrome independently of individual-level SES [47].

A number of limitations affect the inferences of this study. We were unable to determine length of time at reported residence and thus cannot evaluate duration of neighborhood “exposure.” We do not know when neighborhood factors are most likely to contribute to cancer outcomes [43], nor do we know much what period of time is required for a particular neighborhood exposure to affect the biology of disease [48]. We began our investigation at the point of CaP diagnosis. This allowed us to evaluate education consistently in all cases. Another limitation of our study is the fact that the cut-points between more and less advantaged neighborhoods are arbitrary and are dependent upon our sample characteristics. We may also be limited by the “intersection of racial and SES segregation,” in which relatively few AA live in the least deprived areas and few EAs live in the most deprived areas [49]. Thus, study participants are not randomly allocated into census tracts. However, we adjusted for race, age, and census tract in multivariable analysis. We also were not able to detect effects for the group that may be at highest risk: low-SES individuals residing in high-SES neighborhoods. As in other studies, this category was represented by the smallest sample size [22].

The present was ascertained from a tertiary care center, so the external validity of the results may be limited to similar hospital settings. Some cases travel from long distances to receive treatment, which means that this is also a patient group that is educated about healthcare options and has ability to travel for health care. This is also a group that tends to have medical insurance or other means of financing care. Our sample was primarily comprised of low-stage CaP cases (75 %) and was more educated than the general US population (68 % of our sample had a bachelor’s degree, and our median for percent of college-educated residents in the surveyed census tracts was 37 vs. 29 % of the US male population with college degrees). Replication studies with diverse patient populations will add external validity to the results of this study.

We chose to use census tracts as our “neighborhood” variable for this study. Although we could have used other administrative units (census blocks or zip codes), we used the most commonly utilized unit of analysis to increase comparability with other studies. Census tract boundaries are intended to combine individuals that tend to be similar with regard to social and economic characteristics. Census tracts are one of the preferred area-based units to use when attempting to capture economic deprivation. They are meaningful across regions and over time and easily understood/defined [25, 50]. Future studies may examine how cross-level effects vary by administrative unit.

## Conclusions

The effects of neighborhood characteristics on the health of older men have been poorly studied. This project identified CaP cases and communities at highest risk of obesity, advanced cancer and poor treatment outcomes. Obese men are a high-risk group for poor prostate cancer outcomes, but not all obese men carry the same risk. Eliminating CaP disparities requires enhanced efforts to identify highest risk individuals. Empowering disadvantaged communities to improve aspects of the physical or social environment may be an intervention that can benefit the health of residents for years to come. While the present study does not address the mechanisms underlying the association between obesity and prostate cancer aggressiveness, research involving obese prostate cancer cases may suggest new approaches for prostate cancer intervention via weight management, physical activity, targeted screening, CaP education, and novel treatments.
